# Adiponectin Inhibits Oxidative Stress and Tight Junction Protein Loss: Evidence from a Hepatic Encephalopathy Mouse Model and Brain Endothelial Cells

**DOI:** 10.3390/ph19030419

**Published:** 2026-03-04

**Authors:** Dong Jun Song, Seol Won Jeong, Seoyeon Ahn, Danbi Jo, Che-Hun Jung, Jiwoun Park, Sangjun Lee, Juhyun Song

**Affiliations:** 1 Department of Anatomy, Chonnam National University Medical School, Seoyangro 264, Hwasun 58128, Jeollanam-do, Republic of Korea; rylsong@jnu.ac.kr (D.J.S.); sw4068@jnu.ac.kr (S.W.J.); banny6535@naver.com (S.A.); danbijo@jnu.ac.kr (D.J.); 2 Biomedical Science Graduate Program (BMSGP), Chonnam National University, Seoyangro 264, Hwasun 58128, Jeollanam-do, Republic of Korea; jungch@chonnam.ac.kr (C.-H.J.); jiwoun@jnu.ac.kr (J.P.); 3 Department of Biomedical Science and Engineering, Gwangju Institute of Science and Technology, Buk-gu, Gwangju 61005, Republic of Korea; sleefos24@gist.ac.kr

**Keywords:** adiponectin, hepatic encephalopathy, blood–brain barrier, hyperammonia, brain endothelial cell

## Abstract

**Background/Objectives**: Hepatic encephalopathy (HE) is characterized by hyperammonemia, neuroinflammation, oxidative stress, and blood–brain barrier (BBB) dysfunction, with brain endothelial cells being highly vulnerable to ammonia-induced damage. Adiponectin is a cytoprotective adipokine that may enhance endothelial resilience; however, its specific role under hyperammonemic conditions remains unclear. This study aims to investigate the protective effects of adiponectin on brain endothelial function and BBB integrity. **Methods**: In vivo, male C57BL/6J mice underwent bile duct ligation (BDL) surgery and received daily intraperitoneal adiponectin injections (10 μg/kg/day) for 6 days, starting 5 days post-surgery. On day 11, brain tissues and serum were collected for molecular and cytokine analyses. In vitro, mouse brain endothelial cells (bEnd.3) were pretreated with adiponectin before exposure to ammonia. Assays for tight junction preservation, mitochondrial membrane potential, reactive oxygen species (ROS) generation, and total RNA sequencing were performed. **Results**: In BDL mice, adiponectin increased the expression of the tight junction protein claudin-5 and synaptic marker PSD95 across the cortex, hippocampus, and striatum, while reducing pro-oxidant (Cyp2e1, Cyp4a1) and apoptotic (Caspase-9) markers. In vitro, adiponectin pretreatment maintained tight junction proteins, suppressed inflammatory markers, restored mitochondrial membrane potential, and decreased ROS generation in ammonia-exposed bEnd.3 cells. Transcriptomic profiling revealed that adiponectin modulates stress-related gene expression under hyperammonemic conditions. **Conclusions**: Adiponectin enhances cellular stress resistance and maintains BBB structural integrity under ammonia-induced toxicity. These findings suggest that adiponectin serves as a promising therapeutic target for mitigating neurovascular unit dysfunction in hepatic encephalopathy.

## 1. Introduction

Hepatic encephalopathy (HE) is a neuropsychiatric syndrome associated with acute or chronic liver dysfunction, characterized by cognitive impairment, behavioral changes, motor abnormalities, and, in severe cases, impaired consciousness [1,2,3]. Despite clinical advances, HE remains a major cause of morbidity, reduced quality of life, and health-care burden. Multiple mechanisms underlie HE pathogenesis, including systemic inflammation, metabolic dysregulation, and the accumulation of neurotoxic metabolites [4,5]. Of these, hyperammonemia is a consistently recognized and clinically actionable driver of HE [4]. Increasing evidence suggests that HE results from dysfunction of the neurovascular unit, where cerebral endothelial cells, pericytes, astrocytic end feet, and neurons regulate barrier integrity and maintain brain homeostasis [6,7]. The blood–brain barrier (BBB) is primarily formed by specialized brain microvascular endothelial cells connected via tight junctions and supported by astrocytes and pericytes [8]. Tight junction proteins, including claudins and occludin, regulate paracellular permeability and prevent the passage of circulating toxins and inflammatory mediators. Impaired BBB integrity allows peripheral cytokines and immune signals to readily affect the brain parenchyma, exacerbating neuroinflammation and neuronal dysfunction [8,9]. In liver failure, including HE, circulating factors such as ammonia, bile acids, endotoxin, and inflammatory cytokines contribute to BBB breakdown and endothelial dysfunction [10,11].

Ammonia is primarily produced in the gut and detoxified by the liver via the urea cycle; however, its levels increase significantly during liver dysfunction [12]. Elevated ammonia disrupts brain neurotransmission and cellular metabolism, causing mitochondrial dysfunction, impaired membrane potential, increased reactive oxygen species (ROS) production, impaired glutamate metabolism, and inflammatory signaling activation at the cellular level [12,13,14]. In endothelial cells, mitochondrial stress and oxidative injury destabilize tight junction complexes, promote cytoskeletal remodeling, and trigger cytokine release that collectively weaken BBB function [15]. Therefore, strategies that mitigate ammonia-induced oxidative and mitochondrial stress while preserving tight junction integrity may protect neurovascular function in HE.

Bile duct ligation (BDL) is a well-established experimental model of cholestatic liver injury that induces systemic inflammation and metabolic disturbances relevant to HE [16]. BDL causes broad physiological changes that can affect the brain, including altered circulating metabolites and inflammatory mediators [17]. While numerous HE studies focus on astrocyte swelling and neuronal signaling, BBB dysfunction is increasingly recognized as an early and functionally significant feature of HE pathophysiology [18]. Evaluating BBB-associated proteins, including claudin-5 and occludin, and synaptic markers such as postsynaptic density protein 95 (PSD95), alongside oxidative stress and apoptotic pathways, can elucidate how cholestasis-related systemic insults contribute to brain vulnerability. In this study, the effects of adiponectin on BBB protection were investigated in the BDL mouse model.

Adiponectin, an adipose tissue–derived hormone, regulates metabolism, insulin sensitivity, and anti-inflammation. Adiponectin exerts cellular effects primarily through adiponectin receptor 1 (AdipoR1) and receptor 2 (AdipoR2), activating pathways that influence mitochondrial function, oxidative stress responses, and inflammatory transcription [19]. In vascular biology, adiponectin improves endothelial function and reduces inflammatory activation across multiple conditions [20]. These properties suggest that adiponectin may strengthen BBB integrity and enhance endothelial resilience during systemic inflammation and metabolic stress.

However, the role of adiponectin signaling in brain endothelial cells under hyperammonemic stress and its effect on BBB-associated molecular changes in HE remains unclear. Specifically, whether adiponectin protects tight junction architecture during ammonia exposure, normalizes mitochondrial stress responses and ROS production in brain endothelium, and induces supportive molecular signatures in both in vivo HE models and in vitro hyperammonia conditions remains undefined.

Therefore, this study aims to investigate whether adiponectin regulates brain endothelial function and preserves blood–brain barrier (BBB) integrity under hyperammonemic conditions. To achieve this, systemic parameters, BBB integrity, neuroinflammation, and synaptic plasticity markers were examined in a bile duct ligation (BDL) mouse model of HE. In parallel, brain endothelial cells (bEnd.3) were evaluated under in vitrohyperammonemic conditions to assess inflammatory gene expression, tight junction protein preservation, mitochondrial membrane potential, and ROS production. Furthermore, total RNA sequencing was employed to elucidate the transcriptomic changes and downstream signaling pathways modulated by adiponectin pretreatment under hyperammonemic stress.

## 2. Results

### 2.1. Adiponectin Reduced Pro-Inflammatory Cytokine Levels and Reactive Oxygen Species Generation in the Blood of the Bile Duct Ligation Mouse Model

**Figure 1:**
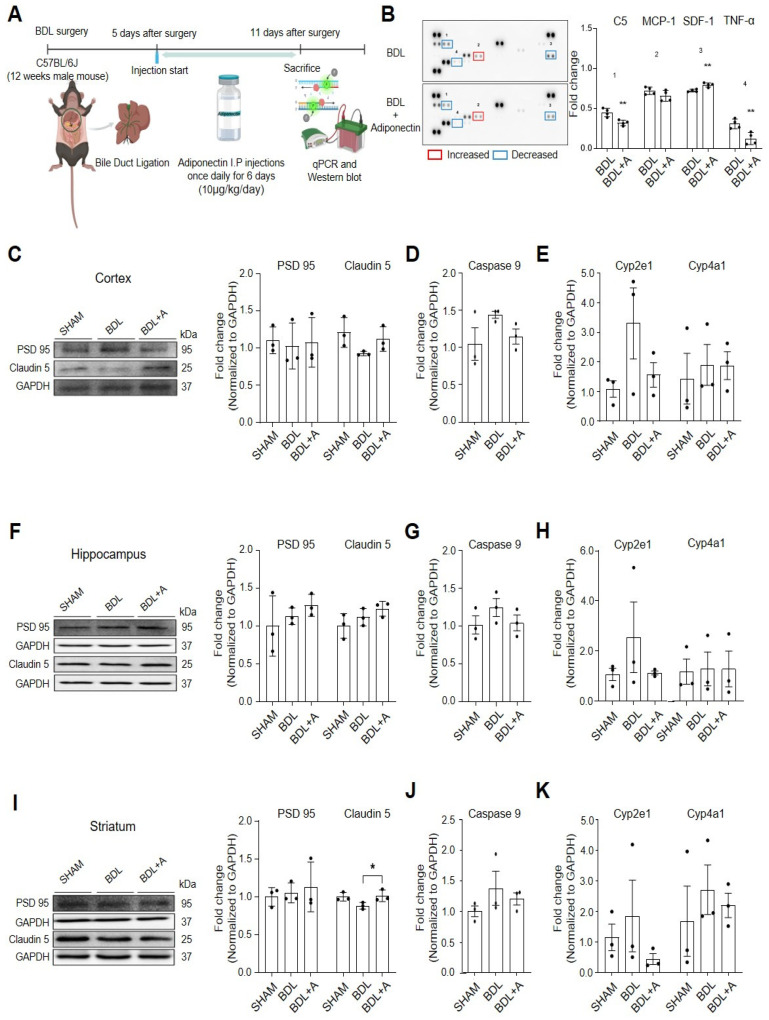
Therapeutic effects of adiponectin administration on systemic parameters and cortical molecular markers in a bile duct ligation (BDL) mouse model. (**A**) Experimental timeline and treatment protocol. Twelve-week-old male C57BL/6J mice underwent a sham operation or BDL. Starting 5 days post-surgery, mice received daily intraperitoneal injections of adiponectin (10 μg/kg) or vehicle for 6 days. Animals were sacrificed on day 11 for molecular analyses. (**B**) Cytokine array analysis of blood from BDL and adiponectin-injected BDL mice. 1:C5, 2:MCP-1, 3:SDF-1, 4:TNF-α. (**C**) Representative Western blots and densitometric quantification of synaptic protein PSD95 and tight-junction protein Claudin5 in the cerebral cortex, with GAPDH as the loading control. (**D**) Relative cortical Caspase-9 mRNA expression, normalized to Gapdh. (**E**) Relative cortical mRNA expression of the ROS-generating enzymes Cyp2e1 and Cyp4a1, normalized to Gapdh. (**F**) Representative Western blots and densitometric quantification of hippocampus PSD95 (synaptic marker) and Claudin-5 (tight junction protein), normalized to GAPDH. (**G**) Relative hippocampal Caspase-9 mRNA normalized to Gapdh. (**H**) Relative hippocampal mRNA expression of ROS-generating enzymes Cyp2e1 and Cyp4a1, normalized to Gapdh. (**I**) Representative Western blots and densitometry of synaptic protein PSD95 and tight junction protein Claudin 5 in the striatum; GAPDH served as a loading control. (**J**) Relative striatal mRNA expression of the apoptosis-related gene Caspase-9, normalized to Gapdh. (**K**) Relative striatal mRNA expression of ROS-generating enzymes Cyp2e1 and Cyp4a1, normalized to Gapdh. Data are shown as mean ± SEM (n = 3 per group). Statistical significance was assessed using an unpaired two-tailed *t*-test with Welch’s correction. * *p* < 0.05, ** *p* < 0.01. Abbreviations: BDL, bile duct ligation; BDL + A, adiponectin injected BDL mouse; PSD95, postsynaptic density protein 95; SHAM, Sham-operated mice were subjected to laparotomy only; ROS, reactive oxygen species; Cyp2e1, cytochrome P450 family 2 subfamily E member 1; Cyp4a1, Cytochrome P450 4A1; C5, complement component C5; MCP-1, monocyte chemoattractant protein-1; SDF-1; stromal cell-derived factor-1; TNF-α, tumor necrosis factor-alpha.

Male C57BL/6J mice underwent either sham or BDL surgery, a common model for hepatic encephalopathy [21]. Adiponectin was administered intraperitoneally (i.p.) at 10 μg/kg/day for 6 days, beginning on day 5 post-surgery, and tissues were collected on day 11 (Figure 1A). Body weight and blood glucose levels were monitored weekly following adiponectin treatment. Body weight progressively decreased in the BDL group and adiponectin-treated BDL mice (Figure 1), while Blood glucose levels remained similar between the two groups throughout the study period. To examine cytokine secretion patterns in BDL mouse blood, cytokine levels were measured using a cytokine array kit (Figure 1B). Adiponectin administration reduced the levels of pro-inflammatory cytokines such as C5, MCP-1, and TNF-α (Figure 1B), while increasing the anti-inflammatory cytokine SDF-1 (Figure 1B). These findings indicate that adiponectin reduces pro-inflammatory cytokine release and ROS generation in BDL mouse blood.

### 2.2. Adiponectin Maintains Tight Junction Proteins, Increased the Expression of Synaptic Plasticity-Related Proteins, and Decreases Inflammatory Responses in Bile Duct Ligation Mouse Brain

Western blotting was performed to evaluate changes in synaptic plasticity density and tight junction proteins in the brain. In the brain cortex, protein expression of the tight junction protein claudin-5 and the synaptic density protein PSD95 was increased in adiponectin-treated BDL mice compared to BDL mice (Figure 1C). Similar increases in PSD95 and claudin-5 were observed in the hippocampus (Figure 1F) and striatum (Figure 1I). In addition, mRNA levels of cell death marker caspase-9 and ROS-related genes, including cyp2e1 and cyp4a1, were analyzed using RT-PCR. In the cortex, adiponectin-treated BDL mice showed reduced mRNA levels of caspase-9, cyp2e1, and cyp4a1 compared to BDL mice (Figure 1D,E). Similar reductions were observed in the hippocampus (Figure 1G,H) and striatum (Figure 1J,K). These findings indicate that adiponectin inhibits cell death signaling while supporting BBB integrity and synaptic stability in the BDL mouse brain.

### 2.3. Effects of Adiponectin on Inflammation, Tight Junction Protein Loss, Mitochondrial Depolarization, and Reactive Oxygen Species Accumulation in Brain Endothelial Cells Under Ammonia Induced Toxicity

**Figure 2:**
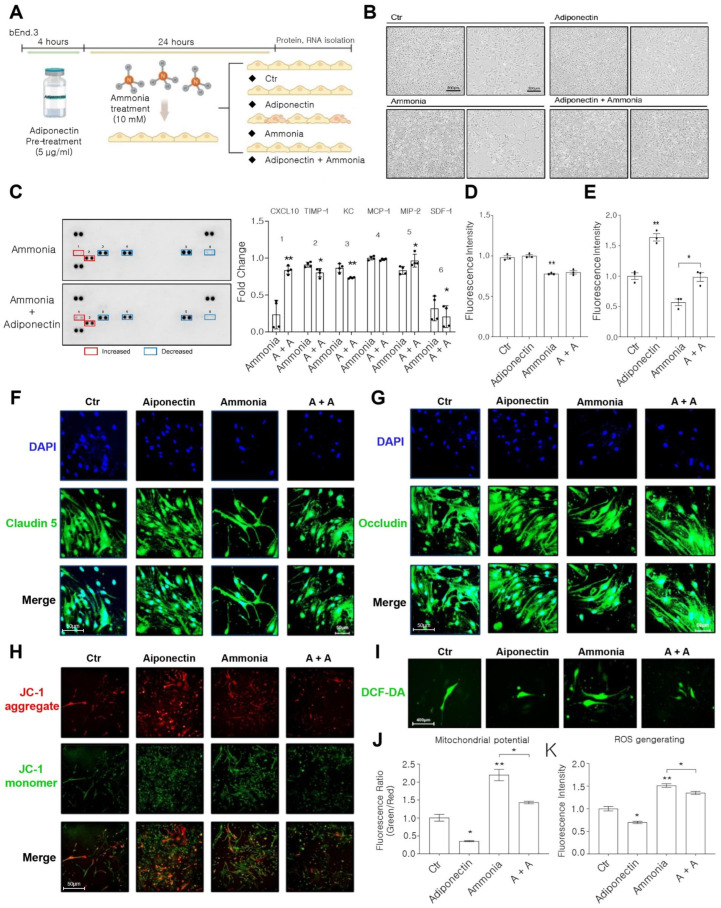
Adiponectin inhibits the secretion of pro-inflammatory cytokines, tight junction protein loss, mitochondrial depolarization, and ROS generation. (**A**) Experimental design for bEnd.3 cells. Cells were pretreated with 10 μg/mL adiponectin for 4 h, subsequently exposed to 10 mM ammonia for 24 h. Experimental groups were Control (Ctr), adiponectin alone, ammonia alone, and adiponectin pretreatment plus ammonia; cells were collected for protein and RNA analyses. (**B**) Virtual cell image for all groups. (**C**) Cytokine array of conditioned media from ammonia- and ammonia + adiponectin–treated cells group. Red and blue boxes indicate relatively increased and decreased cytokine signals, respectively. 1: CXCL10, 2:TIMP-1, 3:KC, 4:MCP-1, 5:MIP-2, 6:SDF-1. (**D**,**E**) immunofluorescence quantification of intensity of tight junction proteins claudin-5 (**D**) and occludin (**E**) in bEnd.3 cells. (**F**,**G**) Representative immunofluorescence images showing claudin-5 (**F**) (green) and occluding, Scale bar: 50 μm (**G**) (green) with nuclear counterstaining (DAPI, blue) across all groups. Scale bars, 50 μm. (**H**) Representative JC-1 images showing mitochondrial membrane potential (aggregates, red; monomers, green) across all groups. Scale bar: 50 μm (**I**). Representative images of intracellular ROS detected by DCF-DA fluorescence (green) in all groups. Scale bar, 400 μm. (**J**) Mitochondrial membrane potential quantified as the JC-1 aggregate/monomer fluorescence ratio across groups. (**K**) ROS levels were quantified using DCF-DA fluorescence intensity in all groups. Data are presented as mean ± SEM of three independent experiments (n = 3). Statistical significance was assessed using an unpaired two-tailed *t*-test with Welch’s correction (* *p* < 0.05, ** *p* < 0.01). Abbreviations: CXCL10, C-X-C motif chemokine ligand 10; TIMP-1, tissue inhibitors of metalloproteinases; KC, keratinocyte-derived chemokine; MCP-1, monocyte chemoattractant protein-1; MIP-2, macrophage inflammatory protein-2; SDF-1, stromal cell-derived factor-1; ctr, control group. No treatment group. A + A, adiponectin pretreated ammonia-exposed bEND.3 cell group; DAPI, 4′,6-diamidino-2-phenylindole (blue color); JC-1 aggregate (red color); JC-1 monomer (green color) DCF-DA, 2′,7′-dichlorofluorescin diacetate; ROS, reactive oxygen species.

To directly investigate the effect of adiponectin on brain endothelial cells in vitro HE conditions, bEnd.3 cells were cultured under hyperammonia conditions (Figure 2A). bEnd.3 cells were pretreated with adiponectin (10 µg/mL) for 4 h before exposure to ammonia (10 mM) (Figure 2A). Figure 2B showed that ammonia-treated bEnd.3 cells exhibited morphological changes associated with cell damage, which were less pronounced in the adiponectin-pretreated group. Adiponectin pretreatment reduced the expression of pro-inflammatory cytokines, including TIMP-1, KC, and MCP-1, while increasing CXCL10 and MIP-2 expression in ammonia-treated bEnd.3 cells (Figure 2C). Immunocytochemistry data showed that adiponectin pretreatment maintained the expression levels of tight junction proteins, including claudin-5 (Figure 2D,F) and occludin, in ammonia-treated bEnd.3 cells (Figure 2E,G). Additionally, mitochondria membrane potential in bEnd.3 cells were assessed using a commercial kit (Figure 2H,J). JC-1 aggregate red fluorescence intensity was higher in adiponectin-pretreated, ammonia-exposed bEnd.3 cells than in ammonia-treated bEnd.3 cell group (Figure 2H,J). Intracellular ROS generation in bEnd.3 cells, was performed using the DCF-DA assay (Figure 2I,K). Collectively, these findings suggest that adiponectin modulates brain endothelial cell function under ammonia toxicity by regulating inflammatory markers, excessive ROS generation, and mitochondrial dysfunction.

### 2.4. Adiponectin Regulates the Expression of Pro-Inflammatory Cytokines and Antioxidant Genes in Brain Endothelial Cells

**Figure 3:**
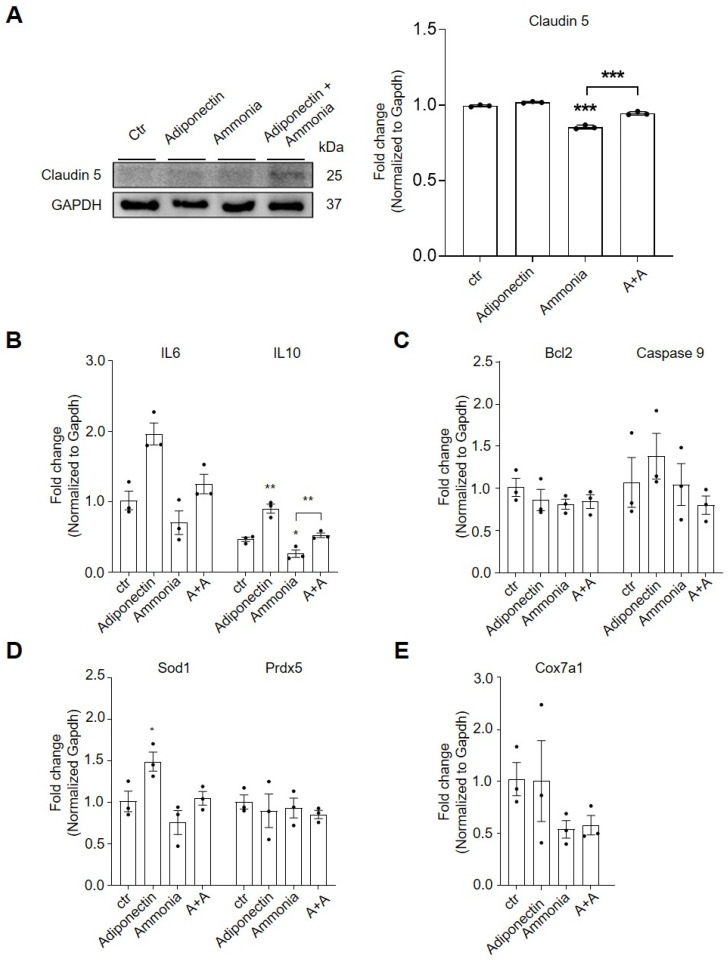
Adiponectin modulates tight junction proteins, and inflammatory cytokines in bEND.3 cells under hyperammonia condition. (**A**) Representative western immunoblots and densitometric claudin 5 protein levels in bEnd.3 cells across all groups. Protein signals were normalized to GAPDH. (**B**–**E**) qPCR analysis measured mRNA expression of (**B**) anti-inflammatory cytokine IL-10 and pro-inflammatory cytokine, (**C**) cell survival marker Bcl2 and apoptotic marker caspase9, (**D**) antioxidant gene Sod1 and Prdx5, (**E**) mitochondrial functional marker Cox7a1 were measured using qRT-PCR analysis across all groups. mRNA levels were normalized to GAPDH. Data are presented as mean ± SEM of three independent experiments (n = 3). Statistical significance was assessed using an unpaired two-tailed *t*-test with Welch’s correction (* *p* < 0.05, ** *p* < 0.01, *** *p* < 0.001). Abbreviations: IL-6, interleukin-6; IL-10, interleukin-10; Bcl2, B-cell lymphoma 2; Sod1, superoxide dismutase 1; Prdx5, peroxiredoxin 5; Cox7a1, Cytochrome C Oxidase Subunit 7A1; Ctr, control group; no treatment group; A + A, adiponectin pretreated ammonia exposed bEND.3 cell group.

We examined the protein levels tight junction protein claudin 5 in adiponectin-treated bEnd.3 cells under hyperammonemic conditions using Western blot analysis (Figure 3A). In ammonia-treated bEnd.3 cells, adiponectin increased claudin-5 protein levels in ammonia-treated bEnd.3 cells compared to untreated cells (Figure 3A). Figure 3B showed that adiponectin treatment increased the mRNA level of the pro-inflammatory IL-6 and anti-inflammatory cytokine IL-10 in ammonia-treated bEnd.3 cells. Analysis of Bcl-2 (cell survival) and Caspase-9 (apoptosis) mRNA expression revealed a protective trend of adiponectin against ammonia-induced cell death; however, the changes were not statistically significant (Figure 3C). Adiponectin treatment increased mRNA levels of superoxide dismutase 1 (Sod1) as an antioxidant gene in ammonia-treated bEnd.3 cells (Figure 3D). Ammonia treatment reduced mRNA levels of cytochrome c oxidase subunit 7A1 (Cox7a1) gene, supporting mitochondrial function, such as adenosine triphosphate (ATP) production in bEnd.3 cells (Figure 3E). Adiponectin treatment showed no significant changes in this marker (Figure 3E). Figure 3 showed that adiponectin maintained claudin-5 expression through AdipoR1 and AdipoR2, reduced pro-inflammatory cytokine levels, and increased antioxidant gene Sod1 in ammonia-treated bEnd.3 cells. While adiponectin did not fully reverse ammonia-induced suppression of IL10 and Sod1 mRNA, it effectively maintained Claudin-5 levels. These findings suggest that adiponectin may exert its protective effects mainly through post-translational regulation or alternative signaling pathways, including G protein–coupled receptors (GPCRs), rather than by reversing transcriptional deficits.

### 2.5. Transcriptomic Alterations by Adiponectin Are Associated with the Modulation of Ammonia-Induced Stress Signaling in Brain Endothelial Cells

**Figure 4:**
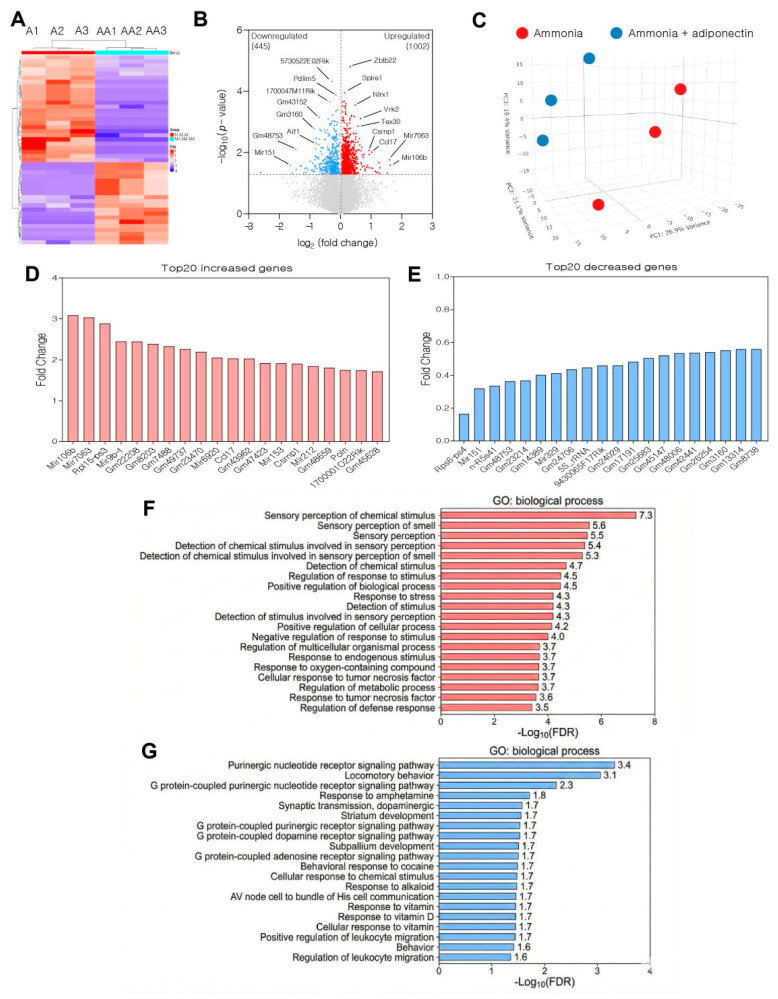
Adiponectin pretreatment modulates transcriptomic profiles in ammonia-exposed brain endothelial cells. (**A**) Heatmap of hierarchically clustered differentially expressed genes (DEGs) illustrating distinct expression patterns between ammonia-treated (A1, A2, A3) and adiponectin-pretreated + ammonia (AA1, AA2, AA3) groups. (**B**) Volcano plot illustrating DEGs distribution. Red dots represent upregulated genes, and blue dots represent downregulated genes in the AA (Adiponectin+ ammonia) group compared to the A (ammonia) group (*p* < 0.05). (**C**) PCA plot showing distinct transcriptomic profiles between ammonia and adiponectin +ammonia groups. (**D**) Bar graph of the top 20 significantly upregulated genes in the adiponectin +ammonia group (ranked by fold change). (**E**) Bar graph of the top 20 most significantly downregulated genes in the adiponectin + ammonia group. (**F**) GO enrichment analysis of the top 800 upregulated genes (*p* < 0.05). The most enriched biological processes include chemical-sensing and GPCR signaling. (**G**) GO enrichment analysis of the top 800 downregulated genes (*p* < 0.05). The analysis reveals a significant suppression of purinergic nucleotide receptor signaling pathways. Abbreviations: A, ammonia treatment group; AA, adiponectin + ammonia treatment group.

To investigate the molecular mechanisms underlying the protective effects of adiponectin against ammonia toxicity, we performed RNA sequencing (RNA-seq) on bEnd.3 cells. We compared the transcriptomic profiles of ammonia-exposed cells (A groups) to those of cells pretreated with adiponectin before ammonia exposure (AA groups). Principal component analysis (PCA; Figure 4C) and hierarchical clustering heatmap (Figure 4A) revealed distinct transcriptomic signatures between the groups, indicating that adiponectin pretreatment globally alters gene expression under hyperammonemic conditions (Figure 4A,C). Figure 4A,B show a clear distinction between the two groups (Figure 4A,C). The volcano plot (Figure 4B) identified key genes—including Pdlim5, Aif1, Gm43152, Gm3160, Gm48753, 5730522E02Rik, 1700047M11Rik, Mir151, Zbtb22, Spire1, Nlrx1, Vrk2, Tex30, Csrnp1, Ccl17, Mir7063, and Mir106b—in adiponectin-pretreated bEnd.3 cells under ammonia conditions (Figure 4B). Among significantly altered genes (*p* < 0.05), the top 20 upregulated genes were selected based on fold-change ranking (Figure 4D). The top 20 upregulated genes in adiponectin-pretreated, ammonia-exposed bEnd.3 cells include Mir106b, Mir7063, Rpl15-ps3, Mir9b-1, Gm22208, Gm8203, Gm7488, Gm49737, Gm23470, Mir6920, Ccl17, Gm43962, Gm47423, Mir153, Csmp1, Mir212, Gm48659, Poln, 1700001O22Rik, and Gm45628 (Figure 4D). Adiponectin pretreatment predominantly upregulated microRNAs (Mir106b, Mir7063, Mir9b-1, Mir153, Mir212) and ribosomal pseudogenes, suggesting enhanced post-transcriptional regulation and translational modulation. Upregulation of the immune-modulatory chemokine Ccl17 and Csmp1 (cysteine-serine-rich nuclear protein) suggests potential activation of cytoprotective signaling pathways. Collectively, these changes reflect the role of adiponectin in promoting stress adaptation and immune homeostasis in ammonia-exposed endothelial cells. Among significantly altered genes (*p* < 0.05), the top 20 downregulated genes were selected based on fold change ranking (Figure 4E). The top 20 downregulated genes in adiponectin-pretreated bEnd.3 cell under ammonia exposure included Rps6-ps4, Mir151, n-R5s41, Gm48753, Gm23214, Gm14389, Mir329, Gm24706, 5S_rRNA, 94300650F17Rik, Gm24029, Gm17191, Gm25683, Gm45147, Gm48006, Gm42441, Gm26254, Gm3160, Gm13314, and Gm8738 (Figure 4E). The downregulated genes were mainly pseudogenes (Rps6-ps4), microRNAs (Mir151, Mir329), and ribosomal RNA components (5S_rRNA, n-R5s41), suggesting reduced stress-induced ribosomal dysregulation and noncoding RNA-mediated inflammatory responses (Figure 4E). The reduction in these genes suggests that adiponectin may help normalize aberrant RNA processing and mitigate ammonia-induced cellular dysregulation. This pattern reflects restoration of cellular homeostasis rather than simple gene suppression.

Using a *p* < 0.05 threshold, the top 800 upregulated and 800 downregulated genes in the adiponectin-pretreated group were selected for GO enrichment analysis (Figure 4F,G).

GO enrichment analysis was conducted on the top 800 upregulated genes (*p* < 0.05) ranked by fold change (Figure 4F). GO enrichment analysis of the 800 upregulated genes revealed terms related to sensory perception of chemical stimuli, regulation of responses to stimuli and stress, immune and defense responses, programmed cell death, and positive regulation of cellular processes and differentiation (Figure 4F). Enriched GO terms among upregulated genes indicate that adiponectin may engage adaptive stress response mechanisms, including oxidative stress defense (response to oxygen-containing compounds and stress), immune regulation (regulation of immune system process and cellular response to TNF), and cell survival signaling (regulation of programmed cell death and positive regulation of cellular processes). Functional analysis of upregulated genes revealed significant enrichment of pathways associated with “sensory perception of chemical stimuli” and “GPCR signaling pathways” (Figure 4F). This suggests that adiponectin may enhance cell survival, potentially priming intracellular signaling pathways necessary for adaptation to toxic stimuli. Collectively, these pathways indicate that adiponectin pretreatment primes endothelial cells for coordinated protection against ammonia-induced oxidative and inflammatory damage.

Subsequently, GO enrichment analysis was conducted on the top 800 downregulated genes (*p* < 0.05) ranked by fold change (Figure 4G). GO enrichment analysis of the top 800 downregulated genes revealed pathways related to purinergic nucleotide and G protein–coupled receptor signaling, locomotory behavior, vitamin responses, leukocyte migration, amine response, and long-term synaptic potentiation (Figure 4G). The downregulated GO terms predominantly involve neurotransmitter signaling (purinergic nucleotide and G protein–coupled dopamine receptor signaling), locomotory behavior, and synaptic function (positive regulation of long-term synaptic potentiation), suggesting suppression of neuronal-like stress responses in endothelial cells. Downregulation of vitamin D response and leukocyte migration indicates reduced pro-inflammatory endothelial activation. This pattern suggests that adiponectin restores endothelial barrier function by reducing abnormal neuronal signaling and excessive immune cell recruitment caused by ammonia toxicity. Additionally, the downregulated genes were strongly enriched in “purinergic nucleotide receptor signaling pathways” (Figure 4G). Given that hyperammonemia-induced extracellular ATP release and purinergic receptor activation influence neuroinflammation, oxidative stress, and BBB disruption, their downregulation suggests a key protective mechanism. Adiponectin may mitigate ammonia toxicity by downregulating aberrant purinergic signaling, thereby influencing downstream inflammatory and apoptotic pathways.

### 2.6. Adiponectin Influences Molecular Networks and Predicted Transcription Factor Targets Associated with Endothelial Homeostasis

**Figure 5:**
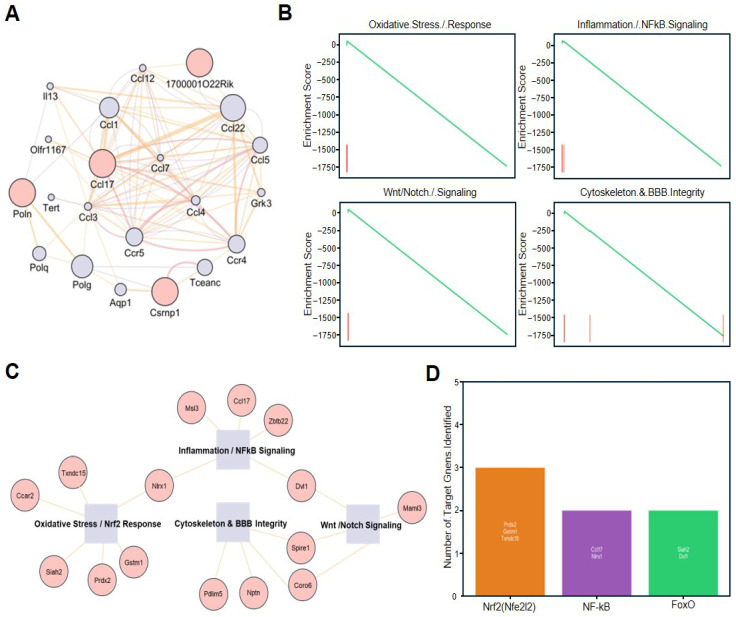
Network Analysis and Transcriptomic Reprogramming Driven by Adiponectin. (**A**) Protein–Protein Interaction (PPI) Network. Visualization of the functional interactome based on the top 20 upregulated genes. Node size correlates with connectivity degree, identifying Ccl17, Ccl22, Ccl5, and Ccr5 as central hub proteins within the adiponectin-modulated landscape. (**B**) Gene Set Enrichment Analysis (GSEA). GSEA enrichment plots for four primary biological clusters: Oxidative Stress Response, Inflammation/NF-κB Signaling, Wnt/Notch Signaling, and Cytoskeleton & BBB Integrity. The downward-sloping profiles indicate a significant negative enrichment trend in these pathways following adiponectin pretreatment under hyperammonemic stress. (**C**) Gene-Pathway Interaction Network. A structured mapping showing the association of specific differentially expressed genes (DEGs) with their respective functional groups. Notably, genes such as Prdx2, Gstm1, and Txndc15 are mapped to the Oxidative Stress/Nrf2 Response, while Ccl17 and Nlrx1 bridge inflammatory signaling and barrier integrity. (**D**) Prediction of Upstream Transcription Factor Targets. Quantitative analysis identifying the number of target genes associated with master regulators. The bar graph highlights the identification of downstream targets for Nrf2 (Nfe2l2), NF-κB, and FoxO, with representative genes (e.g., Prdx2, Gstm1, Siah2) listed within each regulatory category. Abbreviations: A, Ammonia group, AA, Adiponectin + Ammonia group, BBB: Blood–Brain Barrier, Ccl: C-C Motif Chemokine Ligand (e.g., Ccl17, Ccl22, Ccl5), Ccr: C-C Motif Chemokine Receptor (e.g., Ccr5, Ccr4), DEGs: Differentially Expressed Genes, FoxO: Forkhead Box O, GSEA: Gene Set Enrichment Analysis, Gstm1: Glutathione S-Transferase Mu 1, NF-κB: Nuclear Factor-kappa B, Nrf2 (Nfe2l2): Nuclear Factor Erythroid 2-Related Factor 2, PPI: Protein–Protein Interaction, Prdx2: Peroxiredoxin 2, Siah2: Siah E3 Ubiquitin Protein Ligase 2, Txndc15: Thioredoxin Domain Containing 15.

To elucidate the systemic connectivity of the adiponectin-induced transcriptomic changes, we performed a comprehensive network and pathway analysis on the identified DEGs. First, a protein–protein interaction (PPI) network was constructed to visualize the functional clusters among the most significantly top 20 upregulated genes. This analysis revealed a highly interconnected module centered on immune-modulatory chemokines, where Ccl17, Ccl22, Ccl5, and Ccr5 emerged as core hubs, suggesting that adiponectin predominantly modulates the endothelial secretome to manage the local inflammatory environment (Figure 5A). To assess the global impact of adiponectin on biological functions, Gene Set Enrichment Analysis (GSEA) was conducted across several critical domains. The results demonstrated a robust negative enrichment in gene sets related to Oxidative Stress Response, Inflammation/NF-κB Signaling, Wnt/Notch Signaling, and Cytoskeleton & BBB Integrity, confirming that adiponectin pretreatment effectively suppresses the broad stress-related molecular signatures typically triggered by ammonia exposure (Figure 5B). Further integration of these findings through a gene-pathway association network revealed specific molecular links; for instance, cytoprotective genes like Prdx2 and Gstm1 were strategically positioned within the Nrf2-mediated antioxidant response, while hub genes like Ccl17 provided a link between inflammatory signaling and the maintenance of the cytoskeletal architecture required for BBB stability (Figure 5C). Finally, we performed an upstream regulator prediction analysis to identify the master transcription factors potentially associated with these protective effects. This analysis identified a significant number of target genes specifically regulated by Nrf2 (Nfe2l2), NF-κB, and FoxO. Notably, the Nrf2 pathway showed the highest number of identified targets—including Prdx2, Gstm1, and Txndc15—suggesting that the predicted involvement of the antioxidant response may be a primary mechanism through which adiponectin mitigates hyperammonemic damage in brain endothelial cells (Figure 5D).

## 3. Discussion

In this study, we showed that adiponectin was associated with protective effects in a BDL-induced mouse model of hepatic encephalopathy (HE) and in ammonia-exposed brain endothelial cells. Our findings suggest that adiponectin may mitigate systemic inflammatory responses and help maintain the expression of BBB-associated tight junction proteins and synaptic-associated markers, while also showing associations with modulated oxidative stress, mitochondrial function, and transcriptomic profiles in brain endothelial cells. Systemic inflammation acts synergistically with hyperammonemia to promote cerebral edema and cognitive dysfunction in HE [22]. Importantly, the BDL model induces broad cholestatic injury and systemic alterations beyond isolated hyperammonemia, meaning the observed in vivo effects likely reflect a response to generalized hepatic failure rather than ammonia toxicity alone. In the BDL mouse model, adiponectin treatment reduced circulating pro-inflammatory mediators, including TNF-α, MCP-1, and C5, which have been implicated in endothelial tight junction disruption, BBB dysfunction, and leukocyte infiltration into the brain parenchyma [23,24,25,26]. We also observed increased SDF-1, a chemokine that has been associated with tissue repair and context-dependent anti-inflammatory signaling in certain neuropathological settings [27,28]. In contrast, in ammonia-exposed endothelial cells, adiponectin pretreatment was associated with increased expression of CXCL10 and MIP-2 despite an overall reduction in several inflammatory markers. This differential pattern should be interpreted cautiously. Rather than indicating a uniformly suppressive anti-inflammatory effect, it may reflect a context-dependent immunomodulatory response in the local endothelial microenvironment. Because CXCL10 and MIP-2 can participate in leukocyte signaling, endothelial stress responses, and tissue repair, their upregulation may be related to adaptive endothelial signaling under ammonia stress. However, the present study was not designed to define the precise mechanism underlying this selective chemokine regulation. Overall, these findings suggest that adiponectin may modulate systemic and local inflammatory responses in distinct ways rather than acting as a nonselective suppressor of immune signaling [29,30,31].

The structural integrity of the BBB is crucial for inhibiting neurotoxins, including ammonia and inflammatory cytokines, from entering the CNS [32]. In the present study, adiponectin treatment was associated with preservation of the tight junction proteins Claudin-5 and Occludin in the cortex, hippocampus, and striatum of BDL mice, as well as in ammonia-treated bEnd.3 cells [20,33]. This finding is relevant because reduced Claudin-5 expression is considered an early feature of BBB disruption in HE [6]. In addition, preservation of BBB-related proteins was accompanied by maintenance of PSD-95, a postsynaptic density protein involved in synaptic organization and plasticity. Together, these observations are consistent with the possibility that adiponectin supports neurovascular unit stability by preserving endothelial barrier-associated proteins and maintaining a microenvironment that is more favorable for neuronal homeostasis [34,35,36].

In addition to its association with endothelial protection, adiponectin may also influence glymphatic function. Glymphatic clearance depends on intact vascular architecture, preserved perivascular space integrity, and coordinated astrocytic function [37,38]. Because endothelial dysfunction and systemic inflammation are major features of HE, impairment of glymphatic efficiency may plausibly contribute to disease progression. In this context, preservation of endothelial tight junctions and reduction in inflammatory stress by adiponectin may indirectly support glymphatic clearance. However, this interpretation remains hypothetical, as glymphatic function was not directly assessed in the present study. Future studies evaluating glymphatic markers, including aquaporin-4 (AQP4) polarization, will be necessary to determine whether this pathway contributes to the observed protective effects.

At the cellular level, our in vitro findings suggest that adiponectin may counter ammonia-induced endothelial injury which is associated with reducing oxidative stress and stabilizing mitochondrial function. Ammonia toxicity has been linked to mitochondrial permeability transition, reactive oxygen species (ROS) generation, and endothelial cell injury [12,39,40]. In our experiments, adiponectin pretreatment reduced ROS accumulation and was associated with restoration of mitochondrial membrane potential in ammonia-exposed bEnd.3 cells. These changes were accompanied by downregulation of Cyp2e1 and Cyp4a1, which encode cytochrome P450 enzymes that can contribute to oxidative stress under hyperammonemic conditions [41,42,43] and by upregulation of the antioxidant gene Sod1 [24,44]. Since CYP2E1-derived ROS has been implicated in mitochondrial damage, calcium dysregulation, inflammasome activation, and apoptosis-related signaling [45,46], the observed transcriptional changes are consistent with a shift toward a less oxidative cellular state.

RNA sequencing further suggested that adiponectin is associated with a broader stress-response profile in ammonia-exposed endothelial cells. Adiponectin pretreatment was associated with increased expression of several microRNAs, including miR-106b, miR-7063, miR-9b-1, and miR-153. Because microRNAs regulate networks involved in inflammation, oxidative stress, and cell survival [47,48], these findings raise the possibility that adiponectin may be involved in coordinated post-transcriptional regulation under ammonia stress. For example, miR-106b has been linked to cell-cycle regulation and anti-apoptotic signaling [49], which is broadly consistent with the lower Caspase-9 expression observed under ammonia-stressed conditions in the presence of adiponectin pretreatment. Among the upregulated genes, Ccl17 may be relevant to immune modulation [50,51], while Csrnp1 (also known as Axud1) has been implicated in cell survival-related processes [52]. Conversely, among the downregulated transcripts, miR-151 has been associated with apoptosis-related responses in inflammatory injury models [53]. Although these associations are biologically suggestive, the present study does not establish direct causal relationships between these specific transcripts and the protective effects of adiponectin.

Gene Ontology analysis supported this interpretation by showing enrichment of pathways related to the response to oxygen-containing compounds and the regulation of programmed cell death among upregulated genes. These transcriptomic findings are consistent with the biochemical data indicating reduced oxidative stress and improved mitochondrial stability. At the same time, analysis of downregulated genes suggested an association with the suppression of signaling categories related to purinergic receptor signaling and other stress-associated pathways. Under hyperammonemic conditions, aberrant extracellular ATP signaling may contribute to endothelial dysfunction, intracellular calcium dysregulation, inflammasome activation, and BBB impairment. Therefore, the downregulation of these signaling programs may be consistent with preservation of endothelial homeostasis. However, these pathway-level findings should be interpreted as associative rather than definitive mechanistic proof.

Notably, although ammonia reduced Cox7a1, a gene involved in mitochondrial oxidative phosphorylation [54], adiponectin did not significantly reverse this decrease. Nevertheless, adiponectin was still associated with restoration of mitochondrial membrane potential. This may indicate that the protective effect of adiponectin does not require normalization of all ammonia-responsive metabolic genes, but may instead involve stabilization of mitochondrial membrane dynamics and reduction in oxidative stress [55]. Taken together, our findings suggest that adiponectin may exert protective effects in HE through the potential modulation of systemic inflammation, the expression of endothelial barrier proteins, oxidative stress responses, and associative endothelial transcriptomic profiles. These observations support the potential relevance of adiponectin signaling in the regulation of BBB dysfunction under hyperammonemic conditions. Further mechanistic studies, including targeted gain- and loss-of-function approaches, will be necessary to define the specific molecular pathways that mediate these effects more precisely.

## 4. Materials and Methods

### 4.1. Experimental Animals and Study Design

Male C57BL/6J mice, aged 12 weeks, were used in this study. All animals were maintained under controlled environmental conditions with a standard 12 h light/dark cycle and provided ad libitum access to food and water. All experimental procedures involving animals were approved by the Institutional Animal Care and Use Committee of Chonnam National University (Protocol No. CNU IACUC-H-2025-67) and conducted following the “96 Guidance for Animal Experiments.” Hepatic injury was induced in mice via BDL surgery. The mice were randomly divided into three experimental groups (n = 3 per group). (1) sham-operated control group, (2) BDL group treated with vehicle (saline), and (3) BDL group treated with adiponectin. Following a recovery period, the therapeutic effects of adiponectin were evaluated. Drug administration began on day 5 post-surgery. Mice in the treatment group received intraperitoneal (i.p.) injections of adiponectin at a dose of 10 μg/kg once daily for 6 consecutive days. The sham control group received an equivalent volume of saline. Throughout the experimental period, physiological parameters, including body weight and blood glucose levels, were monitored to assess the general health and metabolic status of the animals. On day 11 post-surgery, all animals were anesthetized with isoflurane for tissue collection. Blood samples were obtained, while brain tissues were rapidly dissected. Specific brain regions, including the cortex, hippocampus, and striatum, were isolated on ice for subsequent molecular analyses, such as quantitative real-time polymerase chain reaction (qRT-PCR) and Western blot analysis.

### 4.2. Bile Duct Ligation Surgery

Male wild-type C57BL/6J mice, aged 12 weeks, were purchased from Koatech (Koatech, Pyeongtaek, Republic of Korea). Housing conditions were as described in Section 2.1. All animals were maintained under controlled conditions with a 16 h light/8 h dark cycle, an ambient temperature of 23 °C, and 60 ± 10% relative humidity, with ad libitum access to standard chow and water. To establish the hepatic encephalopathy (HE) model, mice were randomly assigned to the sham-operated group or the BDL group. Anesthesia was induced via intraperitoneal injection of 2,2,2-tribromoethanol/2-methyl-2-butanol (avertin; 0.2 mg/g body weight; Sigma-Aldrich, St. Louis, MO, USA). Subsequently, the BDL procedure was performed using 5-0 silk sutures under maintenance anesthesia with 1.5–2.0% isoflurane delivered in an air–oxygen mixture. BDL surgery mouse technique is a common mouse model for studying HE [21,56]. In sham-operated mice, the bile duct was exposed but not ligated. All experimental analyses were conducted on day 11 post-surgery.

### 4.3. Cloning of the Adiponectin (ADIPOQ) Gene

The Adipoq gene was amplified using polymerase chain reaction (PCR) with a mouse complementary DNA (cDNA) library as a template and the following primers: 5′-GCGCGGATCCGAAGATGACGTTACTACAAC-3′ (forward, BamHI site underlined) and 5′-GAATTCCTCGAGTTATCAGTTGGTATCATGGTAGAGAAG-3′ (reverse, XhoI site underlined). The PCR product was digested with BamHI and XhoI and ligated into the pHIS2 vector, encoding an N-terminal His_6_-tagged adiponectin construct. The recombinant plasmid was transformed into *Escherichia coli* XL1-Blue for propagation and sequence verification, and subsequently into *E. coli* BL21 (DE3) codon plus for protein expression.

### 4.4. Overexpression and Purification of the Adiponectin (ADIPOQ)

*E. coli* BL21 (DE3) cells harboring the recombinant plasmid were cultured in 1 L of LB medium at 37 °C until the optical density at 600 nm reached approximately 0.3. Subsequently, the culture was cooled to 25 °C and incubated for 1 h. Protein expression was induced by adding 0.1 mM isopropyl β-D-1-thiogalactopyranoside and incubating overnight at 25 °C. Cells were collected by centrifugation and resuspended in 50 mM Tris-HCl buffer (pH 8.0) containing 1 mM phenylmethylsulfonyl fluoride. Cells were lysed through sonication, while insoluble debris was removed via centrifugation. The clarified supernatant was loaded onto a Ni-NTA affinity column and eluted using a linear imidazole gradient (10–500 mM). Fractions containing ADIPOQ were pooled and further purified using anion-exchange chromatography on a Q HP column pre-equilibrated with 50 mM Tris-HCl buffer (pH 8.0) and a linear NaCl gradient (0–1 M). Protein purity was assessed using sodium dodecyl sulfate–polyacrylamide gel electrophoresis (SDS–PAGE), while protein concentration was determined at 280 nm using a molar extinction coefficient of 33,350 M^−1^·cm^−1^ [57].

### 4.5. Brain Endothelial Cell Culture

The mouse bEnd.3 cell line was obtained from the American Type Culture Collection bEnd.3 (CRL-2299™, ATCC, Manassas, VA, USA). Cells were cultured in Dulbecco’s modified Eagle’s medium (DMEM; high glucose, L-glutamine; ATCC, Manassas, VA, USA) supplemented with 10% fetal bovine serum (FBS; Millipore, Burlington, MA, USA), penicillin (100 U/mL), streptomycin (100 μg/mL), and sodium pyruvate (1 mM; Thermo Fisher Scientific, Waltham, MA, USA) at 37 °C in a humidified atmosphere with 5% CO_2_. Cells were seeded at specific densities depending on the experimental assay and culture plate format (e.g., 2 × 10^4^ to 6 × 10^4^ cells/well in 24-well plates, and 1 × 10^4^ to 2 × 10^4^ cells/well in 96-well plates). Detailed seeding numbers for each specific assay are explicitly described in the respective methodological subsections below. The culture medium was replaced every 2 days. For adiponectin pretreatment, cells were incubated with recombinant adiponectin (10 μg/mL), a concentration selected based on its ability to elicit protective cellular responses without inducing observable cytotoxic effects under the present experimental conditions, for 4 h before exposure to ammonium chloride. Hyperammonemic stress was induced by treating cells with ammonium chloride (NH_4_Cl; Catalog A9434, Sigma-Aldrich, St. Louis, MO, USA) at a final concentration of 10 mM for 24 h.

### 4.6. Quantitative Real-Time Polymerase Chain Reaction

Total RNA was extracted from the cortex, hippocampus, and striatum of C57BL/6J mice using TRIzol reagent (Invitrogen; Thermo Fisher Scientific, Waltham, MA, USA) following the instructions of the manufacturer. RNA concentration and purity were assessed using a NanoDrop spectrophotometer (IMPLEN, Westlake Village, CA, USA). Total RNA was reverse-transcribed into cDNA using random hexamer primers and RevertAid Reverse Transcriptase (Thermo Fisher Scientific, Waltham, MA, USA). Messenger RNA (mRNA) expression levels were measured using qRT-PCR with TOPreal™ SYBR Green qPCR High-ROX PreMIX (RT510M, Enzynomics, Daejeon, Republic of Korea) on a StepOnePlus Real-Time PCR System (Applied Biosystems, Foster City, CA, USA). Relative gene expression was calculated using the comparative Ct (2−ΔΔCt) method and normalized to Gapdh expression. Supplementary Table S1 presents all primer sequences used in this study.

### 4.7. Western Blot Analysis

Mouse brain tissues (cortex, hippocampus, and striatum) and bEnd.3 cells were lysed in ice-cold RIPA buffer (Biosesang, Seoul, Republic of Korea) supplemented with Xpert Protease Inhibitor Cocktail Solution (100×; P3100-001, GenDEPOT, Katy, TX, USA) and Xpert Phosphatase Inhibitor Cocktail Solution (100×; P3200-001, GenDEPOT, Katy, TX, USA). Lysates were incubated on ice for 15 min, followed by centrifugation at 14,000× *g* for 20 min at 4 °C to collect the supernatant. Protein concentrations were determined using the Pierce BCA Protein Assay Kit (#23227, Thermo Fisher Scientific, Waltham, MA, USA). Equal amounts of protein (30 μg per sample) were resolved using 10–12% SDS–PAGE and transferred onto polyvinylidene difluoride membranes (IPVH08100, Merck Millipore, Burlington, MA, USA) pre-activated with absolute methanol (CAS No. 67-56-1, Merck, Darmstadt, Germany). Membranes were blocked in Tris-buffered saline containing 0.1% Tween 20 (TBS-T) supplemented with 5% bovine serum albumin (BSA; A0100-010, GenDEPOT) for 1 h at room temperature and incubated overnight at 4 °C with specific primary antibodies (1:1000 dilution) prepared in TBS-T containing 5% BSA. Subsequently, membranes were washed three times with TBS-T and incubated with horseradish peroxidase (HRP)-conjugated secondary antibodies (1:5000 dilution) for 2 h at room temperature. Protein bands were detected using Immobilon Western Chemiluminescent HRP Substrate (WBKLS0500, Millipore) and imaged with the Fusion Solo Imaging System (Vilber Lourmat, Collégien, France). Band intensities were quantified using ImageJ (version 1.54d; National Institutes of Health, Bethesda, MD, USA) and normalized to glyceraldehyde-3-phosphate dehydrogenase (GAPDH, sc-32233, Santa Cruz Biotechnolohy, Dallas, TX, USA) as the loading control. The primary antibodies used were anti-claudin-5 (ab15106, Abcam, Cambridge, UK), anti-postsynaptic density 95 (PSD95, 3409s, Cell Signaling Technology, Danvers, MA, USA).

### 4.8. Cytokine Level Detection

To evaluate cytokine secretion from bEnd.3 cells, conditioned media were collected from the ammonia-treated and adiponectin + ammonia-treated groups and centrifuged to remove cellular debris. To measure circulating cytokine levels in BDL mice, blood samples were collected from the BDL and BDL + adiponectin groups, allowed to clot for 30 min at room temperature, and centrifuged at 2000× *g* for 10 min at 4 °C to obtain serum. Conditioned media and serum samples were analyzed using the Proteome Profiler Mouse Cytokine Array Kit (Panel A; ARY006, R&D Systems, Minneapolis, MN, USA) following the instructions of the manufacturer. Array membranes were blocked with Array Buffer 6 for 1 h at room temperature and incubated overnight at 4 °C with the sample mixture (supernatant or serum diluted in Array Buffer 4). After washing, the membranes were incubated with a biotinylated detection antibody cocktail diluted in Array Buffer 4/6 for 1 h at room temperature, followed by incubation with streptavidin–HRP solution (1×) for 30 min at room temperature. Chemiluminescent signals were detected using the Chemi Reagent Mix provided in the kit and imaged with the Fusion Solo imaging System (Vilber Lourmat, Collégien, France). Spot pixel intensities were measured using ImageJ (version 1.54d; National Institutes of Health, Bethesda, MD, USA) and normalized to the reference control spots for comparative analysis.

### 4.9. Immunocytochemical Analysis

bEnd.3 cells were seeded at a density of 1 × 10^4^ cells per well onto poly-L-lysine-coated coverslips in culture plates. After treatment, cells were fixed with 2% paraformaldehyde diluted in phosphate-buffered saline (PBS) for 15 min at room temperature. Following fixation, cells were washed three times with PBS and incubated overnight at 4 °C with primary antibodies diluted 1:200 in GDB buffer (0.1% gelatin, 0.3% Triton X-100, 16 mM sodium phosphate, 450 mM NaCl, pH 7.4). The following day, cells were washed three times with PBS and incubated with fluorophore-conjugated secondary antibodies diluted 1:200 in GDB buffer for 1 h at room temperature in the dark. Nuclei were counterstained with 4′,6-diamidino-2-phenylindole (DAPI) using a DAPI-containing mounting medium. Images were acquired using a Zeiss LSM 710 confocal microscope (Carl Zeiss, Oberkochen, Germany), while fluorescence intensities were quantified using ImageJ (version 1.54d; National Institutes of Health, Bethesda, MD, USA). The primary antibodies used included anti-claudin 5 (ab15106, Abcam, Cambridge, UK) and anti-occludin (sc-133256, Santa Cruz Biotechnology, Dallas, TX, USA). Secondary antibodies were Alexa Fluor 488-conjugated goat anti-rabbit IgG (A-11008, Invitrogen; Thermo Fisher Scientific, Waltham, MA, USA).

### 4.10. Reactive Oxygen Species Generation Analysis

Intracellular ROS production in bEnd.3 cells were assessed using 2′,7′-dichlorodihydrofluorescein diacetate (DCFH-DA; #35845, Sigma-Aldrich, St. Louis, MO, USA). For fluorescence imaging, cells were seeded in 24-well plates at 2 × 10^4^ cells/well. For quantitative measurements, cells were seeded in 96-well plates at 1 × 10^4^ cells/well. A 10 mM DCFH-DA stock solution was prepared in dimethyl sulfoxide (DMSO) and diluted in serum-free culture medium to a final concentration of 10 μM. After treatment, cells were washed once with PBS and incubated with DCFH-DA working solution (1 mL/well for 24-well plates; 100 μL/well for 96-well plates) at 37 °C for 30 min in the dark. Following two additional PBS washes, fluorescent images were acquired using a Zeiss Axio Observer fluorescence microscope (Carl Zeiss, Oberkochen, Germany) for qualitative analysis, while fluorescence intensity was quantified using a microplate reader (excitation: 485 nm; emission: 535 nm). Fluorescence results were expressed as fold change relative to the control group.

### 4.11. Mitochondrial Membrane Potential Assay

Mitochondrial membrane potential (ΔΨm) in bEnd.3 cells was assessed using the JC-1 mitochondrial membrane potential assay kit (5,5′,6,6′-tetrachloro-1,1′,3,3′-tetraethylbenzimidazolocarbocyanine iodide; ab113850, Abcam, Cambridge, UK). For fluorescence imaging, cells were seeded in 24-well plates at 6 × 10^4^ cells/well. For quantitative analysis, cells were seeded in 96-well plates at 2 × 10^4^ cells/well. Cells were assigned to experimental groups (control, ammonia, adiponectin, and ammonia + adiponectin) and treated under the specified conditions. JC-1 staining solution was prepared by diluting the 1 mM JC-1 stock solution in DMSO to a final concentration of 1 μM in serum-free culture medium. After treatment, cells were incubated with JC-1 working solution (500 μL/well for 24-well plates; 100 μL/well for 96-well plates) at 37 °C for 20 min in the dark. Following incubation, cells were washed twice with the JC-1 dilution buffer provided in the kit. Fluorescence was measured at excitation/emission wavelengths of 540/590 nm for red fluorescence (J-aggregates, indicating high ΔΨm) and 485/530 nm for green fluorescence (J-monomers, indicating low ΔΨm). ΔΨm was calculated as the ratio of red to green fluorescence intensity. Fluorescence intensity was measured using a SpectraMax M3 microplate reader (Molecular Devices, San Jose, CA, USA). Fluorescence images were acquired using a Nikon Eclipse Ts2 fluorescence microscope (Nikon Corporation, Tokyo, Japan) and analyzed using ImageJ (version 1.54g; National Institutes of Health, Bethesda, MD, USA). Data were normalized to the control group and expressed as relative ΔΨm.

### 4.12. RNA Sequencing and Functional Analysis of Changed Genes

Total RNA was extracted from brain endothelial cells both in the ammonia group and adiponectin +ammonia group using TRIzol Reagent (Takara, Nojihigashi, Japan) following the manufacturer’s protocol. Genomic DNA contamination was removed through DNase I treatment (Takara, Nojihigashi, Japan). RNA integrity and quality were assessed using the 2100 Bioanalyzer (Agilent Technologies, Santa Clara, CA, USA). RNA sequencing libraries were prepared using TruSeq Stranded Total RNA Kit (Illumina, San Diego, CA, USA) and sequenced on the NovaSeq 6000 System (Illumina, San Diego, CA, USA). Raw sequence reads were quality-filtered and trimmed using Trimmomatic [58], and the processed reads were aligned to the mouse reference genome (mm10) using the STAR aligner 2.7.11b [59]. Gene expression levels were quantified as fragments per kilobase of transcript per million mapped reads (FPKM) using Cuffnorm with GENCODE annotation (Release M23, GRCm38.p6) [60]. For subsequent analyses, genes with average FPKM values < 1 across all samples or equal to 0 in any sample were excluded to ensure data reliability. Differential expression between ammonia-treated and adiponectin + ammonia-treated brain endothelial cells was assessed using Student’s *t*-test, with *p* < 0.05 considered significant. This criterion identified 800 upregulated and downregulated differentially expressed genes, which were analyzed for functional pathways using Gene Ontology (GO) enrichment through the Molecular Signatures Database (MSigDB v7.5.1) [61]. Pathway enrichment was further examined using the WikiPathways 2024 Mouse gene set library via the Enrichr web tool (https://maayanlab.cloud/Enrichr/, accessed on 1 January 2026) [62,63]. The top 20 upregulated and downregulated genes with *p* < 0.05 were selected based on fold-change values. For the most significantly altered genes in brain endothelial cells following adiponectin treatment, a protein interaction network was constructed using the top 20 genes ranked by fold changes with *p* <0.05. Network analysis was conducted with the GeneMANIA plugin [64] (http://www.genemania.org/plugin/, accessed on 1 January 2026) in Cytoscape [65] (version 3.9.1), enabling visualization of functional relationships among the differentially expressed genes. Databases and web tools (MSigDB, WikiPathways, GeneMANIA, and Enrichr) were accessed for retrieval and analysis between [December 2025] and [January 2026].

### 4.13. Functional Enrichment and Network Analysis

To elucidate the molecular mechanisms underlying the protective effects of adiponectin, we performed a comprehensive bioinformatics analysis on the differentially expressed genes (DEGs). First, Gene Set Enrichment Analysis (GSEA) was conducted to identify biological pathways that were significantly modulated. Genes were ranked based on a metric combining statistical significance and magnitude of change. Enrichment scores were calculated for key gene sets related to oxidative stress response, neuroinflammation, and BBB integrity to assess the global upregulation or downregulation of these functional categories. To visualize the systemic connectivity between DEGs and their associated biological processes, a gene-pathway interaction network was constructed using NetworkX 3.6.1 versionand Matplotlib 3.10 x version in Python. High-ranking DEGs were mapped to their functional annotations, including Wnt/Notch signaling, innate immunity, and cytoskeletal organization. In the resulting network, nodes represented individual genes and pathway terms, while edges indicated functional associations. This topological analysis was used to identify hub pathways that bridge metabolic regulation and neuroprotection.

### 4.14. Prediction of Upstream Regulators

To identify potential master regulators driving the observed transcriptomic changes, we performed an upstream regulator prediction analysis. Transcription factors (TFs) were inferred by analyzing the enrichment of their known target genes within the DEG list. Specifically, we focused on TFs relevant to cellular stress responses, such as Nrf2 (Nfe2l2), NF-κB, and FoxO, to validate whether the adiponectin-mediated protective effects are regulated through these signaling cascades.

### 4.15. Statistical Analysis

Data are presented as mean ± SEM. To ensure clarity regarding sample sizes, for in vitro experiments, n represents exactly three independent biological replicates (distinct experimental preparations, rather than technical replicates). For in vivo experiments, n represents exactly three independent individual mice per group. Owing to the small sample size (n = 3 per group), formal tests of normality were considered underpowered and were interpreted cautiously.

For multi-group comparisons, such as the four-group in vitro experiments, statistical significance was assessed using a one-way analysis of variance (ANOVA) followed by Tukey’s multiple-comparisons test. Comparisons between two independent groups, such as the in vivo BDL versus BDL + A groups, were performed using an unpaired two-tailed Student’s *t*-test, with Welch’s correction applied when variances were unequal. Exact *p*-values are provided in the respective figure legends where possible. A *p* value < 0.05 was considered statistically significant.

## 5. Limitations of the Study

Despite the promising findings of this study, several limitations should be acknowledged. First, there was an important difference in experimental design between the in vitro and in vivo models. In the cell-based experiments, adiponectin was administered as a pretreatment before NH4Cl exposure, whereas in the animal model, adiponectin was administered after HE induction as a therapeutic intervention. Therefore, the in vitro findings primarily reflect a preventive paradigm, while the in vivo findings reflect a treatment paradigm. Because these two experimental settings are not directly equivalent, caution is required when interpreting mechanistic continuity and translational relevance across models. Future studies should directly compare prophylactic and therapeutic administration strategies in both in vitro and in vivo settings to clarify the timing-dependent effects of adiponectin more rigorously.

Second, the relatively small sample size in the in vivo experiments (n = 3 per group) may limit statistical robustness and reduce the generalizability of the observed effects. Although the results were consistent across the measured endpoints, additional studies with larger cohorts will be necessary to confirm the reproducibility and precision of these findings. Furthermore, our evaluation of blood–brain barrier (BBB) preservation relied primarily on surrogate molecular markers, such as the expression of tight junction proteins (e.g., claudin-5 and occludin). Without direct functional permeability assays, such as Evans blue or fluorescent dextran leakage, claims regarding the physical integrity of the barrier remain to be functionally validated in future studies.

Third, the bile duct ligation (BDL) model utilized in this study induces a broad cholestatic injury encompassing multiple systemic alterations, including severe inflammation and metabolic disturbances, rather than isolated hyperammonemia. As we did not directly measure blood ammonia levels or core liver injury parameters (e.g., ALT, AST, bilirubin), the observed neuroprotective effects should be interpreted cautiously as a response to generalized hepatic failure rather than solely ammonia-induced toxicity. Fourth, regarding our methodological approach, the use of *E. coli*-derived recombinant adiponectin without a definitive endotoxin exclusion test (e.g., LAL assay) poses a constraint, although the overall reduction in pro-inflammatory cytokines in vivo strongly suggests that endotoxin interference was not the primary driver of our results. Additionally, while the cytokine array provided valuable insights, it serves as a semi-quantitative screening tool that warrants future validation via ELISA. In addition, several pharmacokinetic and pharmacodynamic barriers must be addressed before clinical translation. The large molecular weight of exogenous adiponectin may limit its ability to cross the intact blood–brain barrier efficiently, posing a challenge for systemic administration in central nervous system disorders. Therefore, small-molecule AdipoR agonists (e.g., AdipoRon) or targeted delivery approaches may represent more feasible therapeutic strategies. Furthermore, the optimal therapeutic window, dosing schedule, and treatment duration remain to be determined, particularly because sustained receptor activation may be required to support endothelial barrier function under chronic pathological conditions such as HE.

Finally, our mechanistic insights, including transcriptomic pathway predictions (e.g., Nrf2 and NF-κB modulation) and potential glymphatic function improvements, rely on associative molecular data and remain largely hypothetical. Future studies should therefore assess glymphatic clearance-related markers, such as astrocytic aquaporin-4 (AQP4) polarization, perform direct causal validations of the predicted signaling pathways, and further investigate the kinetics of AdipoR1/2-mediated downstream signaling as well as specific microRNA targets to better define the therapeutic mechanisms involved.

## 6. Conclusions

In conclusion, our study demonstrates that adiponectin exerts neuroprotective effects by mitigating oxidative stress and preserving BBB-associated tight junction protein in models of hepatic encephalopathy. The pleiotropic mechanisms underlying this protection encompass: (1) reduction in systemic pro-inflammatory cytokines, (2) stabilization of tight junction markers, (3) suppression of mitochondrial ROS through modulation of CYP enzymes and SOD1, and (4) transcriptomic modulating that upregulates protective microRNAs while suppressing aberrant endothelial signaling pathways.

Collectively, adiponectin is associated with a dual molecular response: it suggests the attenuation of detrimental purinergic signaling associated with inflammation [66], while simultaneously highlighting the potential engagement of adaptive GPCR-mediated pathways involved in cellular defense [67,68]. This protective mechanism potentially involves predicted upstream regulators, including the Nrf2-mediated antioxidant response coupled with the suppression of NF-κB-dependent inflammatory cascades. By delineating these central hub genes and transcription factors, our study provides a robust molecular foundation for adiponectin-based therapeutic strategies, highlighting its potential to support neurovascular unit stability and cognitive function in patients with chronic liver failure.

## Data Availability

The original contributions presented in this study are included in the article. Further inquiries can be directed to the corresponding author.

## Supplementary Materials

The following supporting information can be downloaded at: https://www.mdpi.com/article/10.3390/ph19030419/s1, Table S1: The list of PCR primers.

## Abbreviations

A	Ammonia (treatment group in vitro)
AA	Adiponectin + ammonia (treatment group in vitro)
ADIPOQ	Adiponectin gene
ATP	Adenosine triphosphate
BBB	Blood–brain barrier
BDL	Bile duct ligation
BSA	Bovine serum albumin
Ccl	C-C motif chemokine ligand
Ccr	C-C motif chemokine receptor
cDNA	Complementary DNA
CNS	Central nervous system
Cox7a1	Cytochrome c oxidase subunit 7A1
Csrnp1	Cysteine-serine-rich nuclear protein 1 (also known as AXUD1)
Ctr	Control group
DAPI	4′,6-diamidino-2-phenylindole
DCFH-DA/DCF-DA	2′,7′-dichlorodihydrofluorescein diacetate
DEGs	Differentially expressed genes
DMEM	Dulbecco’s modified Eagle’s medium
DMSO	Dimethyl sulfoxide
FBS	Fetal bovine serum
FoxO	Forkhead box O
FPKM	Fragments per kilobase of transcript per million mapped reads
GO	Gene Ontology
GPCR	G protein–coupled receptor
GSEA	Gene set enrichment analysis
Gstm1	Glutathione S-transferase mu 1
HE	Hepatic encephalopathy
HRP	Horseradish peroxidase
IL-6	Interleukin-6
IL-10	Interleukin-10
i.p.	Intraperitoneal
JC-1	5,5′,6,6′-tetrachloro-1,1′,3,3′-tetraethylbenzimidazolocarbocyanine iodide
MCP-1	Monocyte chemoattractant protein-1
miR/Mir	microRNA (e.g., miR-106b, Mir7063, etc.)
mRNA	Messenger RNA
NH_4_Cl	Ammonium chloride
NLRP3	NLR family pyrin domain containing 3 (inflammasome)
Nrf2 (Nfe2l2)	Nuclear factor erythroid 2-related factor 2
NF-κB	Nuclear Factor kappa B
PBS	Phosphate-buffered saline
PCA	Principal component analysis
PCR	Polymerase chain reaction
PPI	Protein–protein interaction
PSD95/PSD-95	Postsynaptic density protein 95
qRT-PCR	Quantitative real-time polymerase chain reaction
RNA-seq	RNA sequencing
ROS	Reactive oxygen species
SDF-1	Stromal cell-derived factor-1
SEM	Standard error of the mean
Siah2	Siah E3 ubiquitin protein ligase 2
Sod1	Superoxide dismutase 1
TBS-T	Tris-buffered saline with Tween 20
TNF-α	Tumor necrosis factor-alpha
Txndc15	Thioredoxin domain containing 15
ΔΨm	Mitochondrial membrane potential

## References

[1] Lu K. (2023). Cellular Pathogenesis of Hepatic Encephalopathy: An Update. Biomolecules.

[2] Lopez-Franco O., Morin J.P., Cortes-Sol A., Molina-Jimenez T., Del Moral D.I., Flores-Munoz M., Roldan-Roldan G., Juarez-Portilla C., Zepeda R.C. (2021). Cognitive Impairment After Resolution of Hepatic Encephalopathy: A Systematic Review and Meta-Analysis. Front. Neurosci..

[3] American Association for the Study of Liver Diseases, European Association for the Study of the Liver (2014). Hepatic encephalopathy in chronic liver disease: 2014 practice guideline by the European Association for the Study of the Liver and the American Association for the Study of Liver Diseases. J. Hepatol..

[4] Lemberg A., Fernandez M.A. (2009). Hepatic encephalopathy, ammonia, glutamate, glutamine and oxidative stress. Ann. Hepatol..

[5] Niknahad H., Mobasheri A., Arjmand A., Rafiei E., Alidaee S., Razavi H., Bagheri S., Rezaei H., Sabouri S., Najibi A. (2023). Hepatic encephalopathy complications are diminished by piracetam via the interaction between mitochondrial function, oxidative stress, inflammatory response, and locomotor activity. Heliyon.

[6] Le Guennec L., Mouri S., Thabut D., Weiss N. (2025). Blood-brain barrier dysfunction in hepatic encephalopathy: Pathophysiology, diagnostic assessment and therapeutic perspectives. Metab. Brain Dis..

[7] Chaganti J., Zeng G., Patil A., Lockart I., Dellalana M., Montagnese S., Brew B., Danta M. (2026). Altered blood-brain barrier permeability is associated with abnormal distant connectivity and regional homogeneity in covert hepatic encephalopathy-A cross-sectional study. Hepatology.

[8] Edwards L., Gonzalez A.I., Thomas K.R., Smirnov D.S., Brenner E.K., Nation D.A., Chang F., Blennow K., Salmon D.P., Galasko D. (2025). Interactive effects of blood-brain barrier breakdown and Alzheimer’s disease biomarker status on cognitive decline in older adults without dementia. Alzheimers Dement..

[9] Contreras J.A., Fujisaki K., Ortega N.E., Barisano G., Sagare A., Pappas I., Chui H., Ringman J.M., Joe E.B., Zlokovic B.V. (2024). Decreased functional connectivity is associated with increased levels of Cerebral Spinal Fluid soluble-PDGFRbeta, a marker of blood brain barrier breakdown, in older adults. Brain Imaging Behav..

[10] Fiaschini N., Mancuso M., Tanori M., Colantoni E., Vitali R., Diretto G., Lorenzo Rebenaque L., Stronati L., Negroni A. (2022). Liver Steatosis and Steatohepatitis Alter Bile Acid Receptors in Brain and Induce Neuroinflammation: A Contribution of Circulating Bile Acids and Blood-Brain Barrier. Int. J. Mol. Sci..

[11] Del Duca F., Napoletano G., Volonnino G., Maiese A., La Russa R., Di Paolo M., De Matteis S., Frati P., Bonafe M., Fineschi V. (2023). Blood-brain barrier breakdown, central nervous system cell damage, and infiltrated T cells as major adverse effects in CAR-T-related deaths: A literature review. Front. Med..

[12] Milewski K., Orzel-Gajowik K., Zielinska M. (2024). Mitochondrial Changes in Rat Brain Endothelial Cells Associated with Hepatic Encephalopathy: Relation to the Blood-Brain Barrier Dysfunction. Neurochem. Res..

[13] Jayakumar A.R., Tong X.Y., Curtis K.M., Ruiz-Cordero R., Abreu M.T., Norenberg M.D. (2014). Increased toll-like receptor 4 in cerebral endothelial cells contributes to the astrocyte swelling and brain edema in acute hepatic encephalopathy. J. Neurochem..

[14] Nasu Y., Kishikawa S., Imai M., Yokoyama N., Iida I., Tabeta K., Terunuma M. (2025). Ammonia reduces glutamine synthetase expression in astrocytes via activation of Hippo-YAP signaling pathways. Commun. Biol..

[15] Zhou Y., Zhou J., Li P., Xie Q., Sun B., Li Y., Chen Y., Zhao K., Yang T., Zhu L. (2019). Increase in P-glycoprotein levels in the blood-brain barrier of partial portal vein ligation /chronic hyperammonemia rats is medicated by ammonia/reactive oxygen species/ERK1/2 activation: In vitro and in vivo studies. Eur. J. Pharmacol..

[16] Ganjalikhan-Hakemi S., Asadi-Shekaari M., Mirshekari T.R., Pourjafaria F., Nozari M. (2025). Impact of agmatine on cerebral astrocyte reactivity, neurodegeneration, and oxidative stress in bile duct-ligated rats. Metab. Brain Dis..

[17] Dhanda S., Gupta S., Halder A., Sunkaria A., Sandhir R. (2018). Systemic inflammation without gliosis mediates cognitive deficits through impaired BDNF expression in bile duct ligation model of hepatic encephalopathy. Brain Behav. Immun..

[18] Ali S.A., Datusalia A.K. (2025). Berberine Inhibits the Disruption of the Blood-Brain Barrier and Glial Cell Activation in a Rat Model of Acute Hepatic Encephalopathy. Phytother. Res..

[19] Zhang Z., Hu C., Li B., Zhong C., Guo L., Su B., Chen H., Xie H. (2025). Adiponectin pathway regulates cerebral metabolic dysfunction and neuroinflammation via the AdipoR1/PI3K/Akt axis in Perioperative Neurocognitive Disorder. BMC Geriatr..

[20] Song J., Choi S.M., Whitcomb D.J., Kim B.C. (2017). Adiponectin controls the apoptosis and the expression of tight junction proteins in brain endothelial cells through AdipoR1 under beta amyloid toxicity. Cell Death Dis..

[21] Claeys W., Van Hoecke L., Geerts A., Van Vlierberghe H., Lefere S., Van Imschoot G., Van Wonterghem E., Ghesquiere B., Vandenbroucke R.E., Van Steenkiste C. (2022). A mouse model of hepatic encephalopathy: Bile duct ligation induces brain ammonia overload, glial cell activation and neuroinflammation. Sci. Rep..

[22] Ntuli Y., Shawcross D.L. (2024). Infection, inflammation and hepatic encephalopathy from a clinical perspective. Metab. Brain Dis..

[23] Tang Y., Li H., Li J., Liu Y., Li Y., Zhou J., Zhou J., Lu X., Zhao W., Hou J. (2018). Macrophage scavenger receptor 1 contributes to pathogenesis of fulminant hepatitis via neutrophil-mediated complement activation. J. Hepatol..

[24] Rhodes K., Wang Y., DeMorrow S., Gurumallesh P. (2025). The Role of Neuroinflammation in the Pathogenesis of Hepatic Encephalopathy. J. Immunol. Res..

[25] Zhou J., Ma S., Feng D., Tian Y., Li L., Guo H., Shi Y., Cui W., Dong J., Hao S. (2025). C5aR1(+) microglia exacerbate neuroinflammation and cerebral edema in acute brain injury. Neuron.

[26] Strecker J.K., Minnerup J., Schutte-Nutgen K., Gess B., Schabitz W.R., Schilling M. (2013). Monocyte chemoattractant protein-1-deficiency results in altered blood-brain barrier breakdown after experimental stroke. Stroke.

[27] Heo J.I., Kim K.I., Woo S.K., Kim J.S., Choi K.J., Lee H.J., Kim K.S. (2019). Stromal Cell-Derived Factor 1 Protects Brain Vascular Endothelial Cells from Radiation-Induced Brain Damage. Cells.

[28] Liepelt A., Tacke F. (2016). Stromal cell-derived factor-1 (SDF-1) as a target in liver diseases. Am. J. Physiol. Gastrointest. Liver Physiol..

[29] Castane H., Jimenez-Franco A., Onoiu A.I., Cambra-Cortes V., Hernandez-Aguilera A., Parada D., Riu F., Zorzano A., Camps J., Joven J. (2025). Dysregulation of the FGF21-Adiponectin Axis in a Large Cohort of Patients with Severe Obesity and Liver Disease. Int. J. Mol. Sci..

[30] Nobili V., Alisi A., Cutrera R., Carpino G., De Stefanis C., D’Oria V., De Vito R., Cucchiara S., Gaudio E., Musso G. (2015). Altered gut-liver axis and hepatic adiponectin expression in OSAS: Novel mediators of liver injury in paediatric non-alcoholic fatty liver. Thorax.

[31] Zhao S., Zhu Q., Lee W.H., Funcke J.B., Zhang Z., Wang M.Y., Lin Q., Field B., Sun X.N., Li G. (2025). The adiponectin-PPARgamma axis in hepatic stellate cells regulates liver fibrosis. Cell Rep..

[32] Belanger M., Asashima T., Ohtsuki S., Yamaguchi H., Ito S., Terasaki T. (2007). Hyperammonemia induces transport of taurine and creatine and suppresses claudin-12 gene expression in brain capillary endothelial cells in vitro. Neurochem. Int..

[33] Lee B., Jo D., Park J., Kim O.Y., Song J. (2024). Gut microbiota and their relationship with circulating adipokines in an acute hepatic encephalopathy mouse model induced by surgical bile duct ligation. Heliyon.

[34] Claeys W., Van Hoecke L., Lefere S., Geerts A., Verhelst X., Van Vlierberghe H., Degroote H., Devisscher L., Vandenbroucke R.E., Van Steenkiste C. (2021). The neurogliovascular unit in hepatic encephalopathy. JHEP Rep..

[35] Che J., Sun Y., Deng Y., Zhang J. (2024). Blood-brain barrier disruption: A culprit of cognitive decline?. Fluids Barriers CNS.

[36] Cheon S.Y., Song J. (2021). The Association between Hepatic Encephalopathy and Diabetic Encephalopathy: The Brain-Liver Axis. Int. J. Mol. Sci..

[37] Iliff J.J., Wang M., Liao Y., Plogg B.A., Peng W., Gundersen G.A., Benveniste H., Vates G.E., Deane R., Goldman S.A. (2012). A paravascular pathway facilitates CSF flow through the brain parenchyma and the clearance of interstitial solutes, including amyloid beta. Sci. Transl. Med..

[38] Jessen N.A., Munk A.S., Lundgaard I., Nedergaard M. (2015). The Glymphatic System: A Beginner’s Guide. Neurochem. Res..

[39] Skowronska M., Zielinska M., Wojcik-Stanaszek L., Ruszkiewicz J., Milatovic D., Aschner M., Albrecht J. (2012). Ammonia increases paracellular permeability of rat brain endothelial cells by a mechanism encompassing oxidative/nitrosative stress and activation of matrix metalloproteinases. J. Neurochem..

[40] Orzel-Gajowik K., Milewski K., Obara-Michlewska M., Ellert-Miklaszewska A., Magiera A., Kwapiszewska K., Zielinska M. (2025). Unraveling Ammonia-Induced Brain Endothelial Senescence: Role of miRNA-183-5p. Antioxid. Redox Signal.

[41] Schattenberg J.M., Czaja M.J. (2014). Regulation of the effects of CYP2E1-induced oxidative stress by JNK signaling. Redox Biol..

[42] Leclercq I.A., Farrell G.C., Field J., Bell D.R., Gonzalez F.J., Robertson G.R. (2000). CYP2E1 and CYP4A as microsomal catalysts of lipid peroxides in murine nonalcoholic steatohepatitis. J. Clin. Invest..

[43] Yu J., Zhu H., Kindy M.S., Taheri S. (2021). Cytochrome P450 CYP2E1 Suppression Ameliorates Cerebral Ischemia Reperfusion Injury. Antioxidants.

[44] Bai Y., Li K., Li X., Chen X., Zheng J., Wu F., Chen J., Li Z., Zhang S., Wu K. (2023). Effects of oxidative stress on hepatic encephalopathy pathogenesis in mice. Nat. Commun..

[45] Pardini C., Vaglini F., Viaggi C., Caramelli A., Corsini G.U. (2008). Role of CYP2E1 in the mouse model of MPTP toxicity. Parkinsonism Relat. Disord..

[46] Golaszewski P., Wawrzyniak A., Klosowicz M., Burbelka A., Balawender K. (2025). Neurotoxicity of Chronic Alcohol Exposure: Mechanistic Insights, Cellular Disruption, and Emerging Therapeutic Strategies. Int. J. Mol. Sci..

[47] Wang J., Xu F., Zhu X., Li X., Li Y., Li J. (2021). Targeting microRNAs to Regulate the Integrity of the Blood-Brain Barrier. Front. Bioeng. Biotechnol..

[48] Sawant H., Sun B., McGrady E., Bihl J.C. (2024). Role of miRNAs in neurovascular injury and repair. J. Cereb. Blood Flow. Metab..

[49] Ivanovska I., Ball A.S., Diaz R.L., Magnus J.F., Kibukawa M., Schelter J.M., Kobayashi S.V., Lim L., Burchard J., Jackson A.L. (2008). MicroRNAs in the miR-106b family regulate p21/CDKN1A and promote cell cycle progression. Mol. Cell Biol..

[50] Song N., Paust H.J., Asada N., Peters A., Kaffke A., Krebs C.F., Panzer U., Riedel J.H. (2024). Targeting Monocyte Derived CCL17 Attenuates Murine Crescentic Glomerulonephritis by Affecting Renal CCR4+ Regulatory T-Cell Recruitment. Am. J. Nephrol..

[51] Shi H., Chen L., Ridley A., Zaarour N., Brough I., Caucci C., Smith J.E., Bowness P. (2020). GM-CSF Primes Proinflammatory Monocyte Responses in Ankylosing Spondylitis. Front. Immunol..

[52] Wang Z., Ge W., Zhong X., Tong S., Zheng S., Xu X., Wang K. (2024). Inhibition of cysteine-serine-rich nuclear protein 1 ameliorates ischemia-reperfusion injury during liver transplantation in an MAPK-dependent manner. Mol. Biomed..

[53] Zhou F., Chen L., Xu S., Si C., Li N., Dong H., Zheng P., Wang W. (2022). Upregulation of miR-151-5p promotes the apoptosis of intestinal epithelial cells by targeting brain-derived neurotrophic factor in ulcerative colitis mice. Cell Cycle.

[54] Feng Y., Xu J., Shi M., Liu R., Zhao L., Chen X., Li M., Zhao Y., Chen J., Du W. (2022). COX7A1 enhances the sensitivity of human NSCLC cells to cystine deprivation-induced ferroptosis via regulating mitochondrial metabolism. Cell Death Dis..

[55] Zhang S., Wu X., Wang J., Shi Y., Hu Q., Cui W., Bai H., Zhou J., Du Y., Han L. (2022). Adiponectin/AdiopR1 signaling prevents mitochondrial dysfunction and oxidative injury after traumatic brain injury in a SIRT3 dependent manner. Redox Biol..

[56] Abshagen K., Konig M., Hoppe A., Muller I., Ebert M., Weng H., Holzhutter H.G., Zanger U.M., Bode J., Vollmar B. (2015). Pathobiochemical signatures of cholestatic liver disease in bile duct ligated mice. BMC Syst. Biol..

[57] Pace C.N., Vajdos F., Fee L., Grimsley G., Gray T. (1995). How to measure and predict the molar absorption coefficient of a protein. Protein Sci..

[58] Bolger A.M., Lohse M., Usadel B. (2014). Trimmomatic: A flexible trimmer for Illumina sequence data. Bioinformatics.

[59] Dobin A., Davis C.A., Schlesinger F., Drenkow J., Zaleski C., Jha S., Batut P., Chaisson M., Gingeras T.R. (2013). STAR: Ultrafast universal RNA-seq aligner. Bioinformatics.

[60] Trapnell C., Roberts A., Goff L., Pertea G., Kim D., Kelley D.R., Pimentel H., Salzberg S.L., Rinn J.L., Pachter L. (2012). Differential gene and transcript expression analysis of RNA-seq experiments with TopHat and Cufflinks. Nat. Protoc..

[61] Liberzon A., Subramanian A., Pinchback R., Thorvaldsdottir H., Tamayo P., Mesirov J.P. (2011). Molecular signatures database (MSigDB) 3.0. Bioinformatics.

[62] Martens M., Ammar A., Riutta A., Waagmeester A., Slenter D.N., Hanspers K., Miller R.A., Digles D., Lopes E.N., Ehrhart F. (2021). WikiPathways: Connecting communities. Nucleic Acids Res..

[63] Xie Z., Bailey A., Kuleshov M.V., Clarke D.J.B., Evangelista J.E., Jenkins S.L., Lachmann A., Wojciechowicz M.L., Kropiwnicki E., Jagodnik K.M. (2021). Gene Set Knowledge Discovery with Enrichr. Curr. Protoc..

[64] Warde-Farley D., Donaldson S.L., Comes O., Zuberi K., Badrawi R., Chao P., Franz M., Grouios C., Kazi F., Lopes C.T. (2010). The GeneMANIA prediction server: Biological network integration for gene prioritization and predicting gene function. Nucleic Acids Res..

[65] Shannon P., Markiel A., Ozier O., Baliga N.S., Wang J.T., Ramage D., Amin N., Schwikowski B., Ideker T. (2003). Cytoscape: A software environment for integrated models of biomolecular interaction networks. Genome Res..

[66] Engel T., Jimenez-Mateos E.M., Diaz-Hernandez M. (2022). Purinergic Signalling and Inflammation-Related Diseases. Cells.

[67] Liu Y., Zhou J., Zhang N., Wu X., Zhang Q., Zhang W., Li X., Tian Y. (2022). Two sensory neurons coordinate the systemic mitochondrial stress response via GPCR signaling in C. elegans. Dev. Cell.

[68] Feng Z., Sun R., Cong Y., Liu Z. (2022). Critical roles of G protein-coupled receptors in regulating intestinal homeostasis and inflammatory bowel disease. Mucosal Immunol..

