# Supplementary material for: Adiponectin Inhibits Oxidative Stress and Tight Junction Protein Loss: Evidence from a Hepatic Encephalopathy Mouse Model and Brain Endothelial Cells
Source: Pharmaceuticals (Basel). 2026 Mar 4;19(3):419. doi: 10.3390/ph19030419 (PMC13029454; doi:10.3390/ph19030419)
Supplement: Supplementary file 1 [file pharmaceuticals-19-00419-s001.zip › Supplementary Table S1.pdf]

Table S1. The list of PCR primers

Mouse		
**Name**	**Forward sequence**	**Reverse sequence**
**Caspase9**	AGCTGGTCACAGACCTTGAGA	CAGCTTCACTACTCTCTGCTCC
**Cyp2e1**	CTTTGCAGGAACAGAGACCA	ATGCACTACAGCGTCCATGA
**Cyp4a1**	CAACTTGCCCATGATCACACA	CATCCTGCAGCTGATCCTTTC
**IL-6**	ACTTCACAAGTCCGGAGAGG	TGCAAGTGCATCATCGTTGT
**IL-10**	AGACCAAGGTGTCTACAAGGC	CCAAGGAGTTGTTTCCGTTAGC
**Bcl2**	GATGACTGAGTACCTGAACCG	CAGAGACAGCCAGGAGAAATC
**Cox7a1**	GGTCCGGTCTTTTAGCTCAT	GGTCCGGTCTTTTAGCTCAT
**Sod1**	ACTCTAAGAAACATGGTGGCCC	TACGGCCAATGATGGAATGCT
**Prdx5**	GAACCGGGAAAGAAGGTGAAC	CCACTCTTCAATCACAAAGACGT
**Gapdh**	CTGCCATCAGGAAGAAACTGG	TGTCTGGATAAGAGAGAGGCC
